# Development of a biodegradable polymer-based implant to release dual drugs for post-operative management of cataract surgery

**DOI:** 10.1007/s13346-024-01604-y

**Published:** 2024-05-02

**Authors:** Nayana E- Subhash, Soumya Nair, Srilatha Parampalli Srinivas, Nagarajan Theruveethi, Sulatha V- Bhandary, BharathRaja Guru

**Affiliations:** 1 Department of Biotechnology, Manipal Institute of Technology, Manipal Academy of Higher Education, Manipal, Karnataka India; 2 Department of Ophthalmology, Kasturba Medical College Manipal, Manipal Academy of Higher Education, Manipal, Karnataka India; 3 Department of Pathology, Kasturba Medical College Manipal, Manipal Academy of Higher Education, Manipal, Karnataka India; 4 Department of Optometry, Manipal College of Health Professions, Manipal Academy of Higher Education, Manipal, Karnataka India

**Keywords:** PLGA implant, Post cataract management, Ocular drug delivery, Dual drug release

## Abstract

Cataract surgery is followed by post-operative eye drops for a duration of 4–6 weeks. The multitude of ocular barriers, coupled with the discomfort experienced by both the patient and their relatives in frequently administering eye drops, significantly undermines patient compliance, ultimately impeding the recovery of the patient. This study aimed to design and develop an ocular drug delivery system as an effort to achieve a drop-free post-operative care after cataract surgery. An implant was prepared containing a biodegradable polymer Poly-lactic-co-glycolic acid (PLGA), Dexamethasone (DEX) as an anti-inflammatory drug, and Moxifloxacin(MOX) as an antibiotic. Implant characterization and drug loading analysis were conducted. In vitro drug release profile showed that the release of the two drugs are correlated with the clinical prescription for post operative eye drops. In vivo study was conducted on New Zealand albino rabbits where one eye underwent cataract surgery, and the drug delivery implant was inserted into the capsular bag after placement of the synthetic intraocular lens (IOL). Borderline increase in the intraocular pressure (IOP) was noted in the test sample group. Slit-lamp observations revealed no significant anterior chamber reaction in all study groups. Histopathology study of the operated eye revealed no significant pathology in the test samples. This work aims at developing the intra ocular drug delivery implant which will replace the post-operative eye drops and help the patient with the post-operative hassle of eye drops.

**Graphical abstract _(created using BioRender.com)_:**

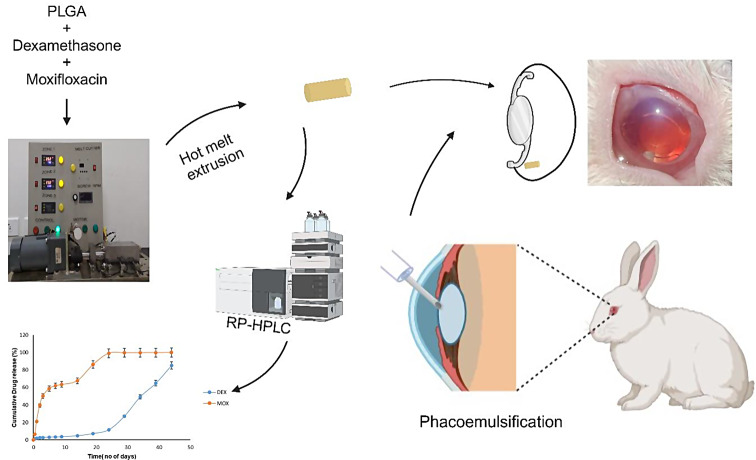

**Supplementary Information:**

The online version contains supplementary material available at 10.1007/s13346-024-01604-y.

## Introduction

The controlled release of ophthalmic drugs has been widely studied over the years. The aim is to provide a platform for drug delivery with reduced frequency and toxicity with limited use of invasive techniques. A cataract is an ocular condition caused because of the clouding of the ocular lens and leads to vision impairment. It is often seen in the elderly population, also known as senile cataract. There are various other causes for cataract like diabetes and prolonged steroid application [1]. It is accounts for over 15.2 million cases representing almost 45% of the population affected by ocular conditions globally and responsible for about 71.2% of cases of vision impairment in India’s population over 50 years of age [2, 3]. The clouding is mostly due to the modifications in the lens proteins which may be due to changes in morphology, biochemistry, or physical changes of the lens [4]. It is easily corrected by cataract surgery which involves the replacement of the natural but opaque lens with an artificial clear one. Because of the possible risk of post-surgery complications, the procedure is followed up with post-operative care where the patient is prescribed to use topical anti-inflammatory and antibiotics for a period of 4–6 weeks in a tapering pattern [5]. A fundamental challenge in ocular drug delivery is the sustained distribution of drugs to the anterior and posterior segments of the eye with low systemic exposure. Patient compliance is often a difficulty in the process of recovery as a majority of the population affected is the elderly [6, 7]. A drawback of topical instillation of drugs is that there is only 1–5% of the applied drug can reach the aqueous humor which is attributed to the various ocular barriers present in the eye [8–10]. It has to be considered that when a huge population is affected by cataract and has to undergo surgery, substantial amount of people are also prone to post-surgery complications. This is especially so in case of developing countries where bulk surgeries are conducted in medical camps to reduce the cost of the surgery and the population affected require a bystander to help instil the eye drops especially in the elderly pateints [11]. There are over 6 million cataract surgeries done in India annully but over 30 thousands of them are reported to have developed severe post-surgery ocular complications [12, 13].

The various physiological barriers prevent and hamper the entry of drugs into the eye leading to low bioavailability of drugs in the ocular tissues. The tear turnover rate is about 16% per minute during the time a person is awake [14], while the aqueous humor turnover is 1-1.5% of its volume per minute [15]. To improve the bioavailability of drugs in ocular tissues there is a need for frequent administration of eye drops. Many carrier based drug delivery systems like contact lenses, nanoparticles, IOLs, implants, and hydrogels have been developed to deliver a drug or a combination of drugs to the eye [7, 16–18]. The use of drug delivery devices can potentially reduce the need for frequent administration of medication and prolong the bioavailability of drugs to treat ocular conditions [19].One of the objectives of this is to replace the post-operative eye drops and make the process “Drop Free” and improve patient compliance. In the treatment of conditions like inflammation and infection, sustained drug release from a biocompatible and biodegradable polymer matrix would be a huge advancement [20]. The drugs are entrapped within the polymer matrix and they are released in slow and sustained manner as the matrix degrades [21]. Poly-lactic-co-glycolic acid (PLGA) is a biodegradable and biocompatible polymer and it is FDA approved for clinical applications [22]. It is a frequently used polymer matrix for drug delivery [23]. Biodegradable and controlled release implants hold significant promise in enhancing the therapeutic effectiveness while minimizing the unwanted side effects across various drug treatments [24]. A variety of procedures are available for the manufacturing of these implants like hot-melt extrusion, compression, melting and molding [25, 26].

Over 20 marketed medications based on PLGA have been approved by the FDA till date [27]. Ozurdex is one of the first intraocular biodegradable implants to be marketed. It is an intravitreal implant contains 0.7 mg of DEX and the effect is exhibited for a period of 6 months [28]. There have been reports on implant migration and increase in intraocular pressure where Ozurdex was used. In some cases, it can also cause corneal edema leading to the need for surgical removal of the implant [29]. Surodex is a biodegradable PLGA implant containing 60 µg of DEX inserted into the anterior chamber to manage post-cataract surgery inflammation. It has a release profile for about 7 days [30]. DEX loaded PLGA implant when inserted into the capsular bag was shown to be efficient in the delivery of drugs to both anterior and posterior segments of the eye for the management of post-surgery inflammation and for uveitis [20].

In the current market, there is a noticeable absence of products offering drop-free post-operative care. Even Surodex, previously considered for this purpose, had the clinical study discontinued. Our study aims to pioneer an ocular drug delivery system tailored for post-operative care following cataract surgery, enabling patients to forgo the use of eye drops [31]. This implant, is the first of its kind which incorporates the two drugs commonly used in post-operative care which is prepared using hot-melt extrusion and positioned within the capsular bag during surgery. This effectively addresses the potential complications such as infection and inflammation after cataract surgery. The capsular bag consists of the posterior capsule of the ocular lens, and a portion of the anterior capsule that houses the IOL [32]. By placing the implant in the capsular bag the possibility of migration into the anterior chamber and contact with corneal endothelium can be avoided thereby mitigating any possible toxicity to the tissue. By integrating the implant insertion into the surgical procedure itself, we aim to seamlessly merge surgery with the treatment. The focus point of using the implant is to act as a preventive measure against possible post-surgery infection and inflammation and for the patient to be able to have a ‘drop-free’ post-operative care. This is an attempt to make an alternative approach to the conventional application of eye drops after cataract surgery. The main goal of this work is to produce the preclinical study data and take the product to the next level for translation. The extruder used in this study was custom made at a local workshop in a frugal way such that it costs less than one twentieth of the commercially available extruders. It was prepared such that it can work with a minimum load of 3 g of material thereby cutting down on material needed per extrusion. We used the polymer PLGA along with the anti-inflammatory drug DEX and the anti-biotic MOX to develop a biodegradable implant by single screw hot-melt extrusion and implant was sterilised using ethylene oxide gas and evaluated using in vivo rabbit model, most suitable model for ocular complications.

## Materials and methods

### Materials

PLGA (50: 50 ester terminated 65-95KDa) was purchased from Nomisma Healthcare Pvt.Ltd (Vadodara, Gujarat, India), DEX was a gift sample from Symbiotec Pharmalab Pvt.Ltd ( Indore, Madhya Pradesh, India), MOX was a gift sample from Shankus Pharmaceuticals (Santej, Gujarat, India), Acetonitrile, Methanol and Hematoxylin were purchased from Merck (India), sodium dihydrogen phosphate and disodium hydrogen phosphate were purchased from Himedia (India), Eosin was purchased from Finar Chemicals (India), Tono-Pen AVIA was purchased from Reichert Technologies.

### Methods

#### HPLC method development

Methods were developed for the simultaneous analysis of DEX and MOX using RP-HPLC (SHIMADZU LC-20AD). The column used was Phenomenex Gemini C18 250 mm and a flow rate of 1.0mL/minute was maintained. DEX was analyzed using a 60:40 ratio of acetonitrile and water as the mobile phase. The analysis of MOX was done using a 35:65 ratio of acetonitrile and 0.05 M phosphate buffer (pH 7.4). For the combination drug analysis, the mobile phase used was acetonitrile and 0.05 M phosphate buffer (pH 7.4) in a 50:50 ratio. DEX was detected at 239 nm while MOX was detected at 295 nm.

Stock solutions of the drugs were prepared in acetonitrile for DEX and phosphate buffer for MOX. For combination the stock solutions were prepared in 1:1 ratio of acetonitrile and phosphate buffer to obtain a concentration of 1 mg/ml. Various dilutions of known concentrations were prepared from the stock solutions and calibration curve was prepared to find out unknown concentration using RP-HPLC from above-mentioned methods..

#### Stability of drug

The stability of the drugs DEX and MOX, individually and in combination was analyzed. 1 mg of each of the drug sets were weighed and dissolved in phosphate buffer of pH 7.4 and kept in a rocker shaker at 37℃ for a period of 30 days. Aliquots were taken at regular time intervals and analyzed using the RP-HPLC method previously described.

#### Implant preparation

The drug loaded polymer implant was prepared using a single screw hot melt extruder. Table 1 shows the dimensions and geometrical design parameters of the extruder. Two stainless steel barrels with adjustable temperature control were used. The first and second barrel were set to 78℃ and 68℃ respectively. The setup was allowed to pre-heat for one hour before the extrusion was conducted. PLGA 50:50 (65-95KDa), DEX, and MOX were taken in the ratio of 10:2:1 and mixed thoroughly before being fed into the extruder. The sample extruded was collected and chopped to obtain implants of approximately 1 mm in length. Implants containing individual drugs and polymer were also prepared. The length, diameter, and weight of each implant were measured and were then stored in a -20℃ freezer till further use.

**Table 1:** Dimensions and geometrical design parameters of the extruder

Symbol	Description	Value
dbarrel	the inner diameter of the barrel	12 mm
hchannel	mean distance from the screw root to the barrel wall	1.2 mm in the beginning and 1 mm at the end
hclearance	clearance between barrel and extruder screw	0.05 mm
wchannel	channel width	4.00 mm
wpitch	the pitch of the extruder screw	6 mm in the beginning and 5.6 mm at the end

#### X-ray diffraction analysis (XRD)

XRD studies were done using Rigaku Miniflex 600 (5th Gen) system. A known dimension of the implant was placed on a sample holder in the chamber for powder X-ray diffraction analysis. XRD patterns were generated for DEX, MOX, PLGA, and dual drug-loaded PLGA implant to understand the uniformity of drug loading in the implant.

#### Scanning electron microscopy (SEM)

SEM analysis was done using EVO MA18 with Oxford EDS(X-act) Model instrument to study the size and surface morphology of the implant. The samples were fixed on aluminum stubs and were gold sputter coated, observed at 100x and 2000x magnification. Analysis was also done on implant samples that were collected at different time intervals after being kept in phosphate buffer at 37^o^ C in rocker shaker kept at 100 rpm to observe the change in surface morphology of the implant with time.

#### Drug loading

Implants in triplicates were placed in vials containing 1 ml of methanol and kept in a rocker shaker for 2 days, after which the samples were centrifuged at 10,000 rpm for 30 min. The supernatant was collected and kept for drying. The dried samples were later resuspended in 1 ml of solvent (1:1 acetonitrile and pH 7.4 0.05 M phosphate buffer mixture) and the concentration of the drug was analyzed using RP-HPLC. To access the batch to batch variation three different sets of the implants were prepared by hotmelt extrusion and analysed for their drug loading.

#### In vitro drug release

Implant samples were taken in triplicates, and each one was kept in 1 ml of 0.1 M phosphate buffer (pH 7.4) in a rocker shaker at 37℃. The samples were collected at defined time intervals and replenished with 1 ml buffer. Samples were collected for a period of six weeks and were analyzed using RP-HPLC. The cumulative drug release concentration was plotted with respect to time (in days).

## In vivo study

### Animals

Institutional Animal Ethics Committee (IAEC/KMC/36/2018) approval was obtained before starting the study. New Zealand albino rabbits of average age 2 years were used, and each animal was placed in an individual cage and supplied with adequate food and water.

### Study design

The rabbits were divided into four groups:

i. Group 1– Normal control; *N* = 4– No Surgery.
ii. Group 2– Positive control; *N* = 4– Phacoemulsification & IOL implantation, topical application of DEX- MOX eye drops, without implant.
iii. Group 3– Sham control; *N* = 4– Phacoemulsification, IOL implantation, plain PLGA implant (without drugs) inserted, topical drops of DEX and MOX were administered regularly as per the guidelines.
iv. Group 4– Test control; *N* = 4– Phacoemulsification, IOL implantation, combination drug implant, no topical drops administered.

The positive control group, sham control group, and test group underwent phacoemulsification surgery on the right eye. The implants were used after ethylene oxide gas sterilization. The implant was placed in the inferior fornix of the capsular bag during surgery after implantation of the IOL. Post-operative eye drops (Miflodex- contains DEX and MOX ) were applied in a tapering pattern for 6 weeks in the positive control and sham control groups on the operated eye. Nepalact (nepafenac: non-steroidal anti-inflammatory agent) was administered thrice a day on the operated eye of the three groups. Nepafenac eye drops are recommended to reduce post operative cystoid macular edema after cataract surgery in humans and is a standard of care. Weekly observations were made using handheld slit-lamp to observe the anterior segment.

### Intraocular pressure (IOP)

A tonopen was used to check the intra ocular pressure. Tonopen is a contact applanation tonometer. The tonometer applanates the cornea on contact using a plunger that moves within a sleeve. Applanation of the cornea moves this tip relative to the plunger and this movement is recorded as a continuous tracing following detection by a transducer. As the forces of corneal resistance are transferred to the sleeve, the applanation pressure concords with the IOP [33]. For taking the IOP, corneal surface was anesthetised using topical proparacaine drops and the rabbit was positioned without exerting pressure on the neck and eyeball. IOP of the operated eye was measured first followed by the fellow eye using the tonopen held like a pen. The tip was cleaned using isopropyl alcohol followed by sterile water between each eye and animal and the disposable ocufilm tip cover was changed after each animal. The digital reading on the screen is recorded as the intra ocular pressure of the eye tested in millimetres of mercury (mm of Hg). Three IOP readings were taken per eye and the average was calculated and tabulated.

### Histopathology

At the end of the observation period, all the 16 animals were sacrificed, and the enucleated eyes were examined for gross morphology and were then preserved in 10% neutral buffered formalin for further processing. Following this the tissues were dehydrated by immersion through different grades of alcohol. Subsequently, the tissues were embedded in paraffin, forming sturdy blocks that were stored at 4 °C until further processing. Utilizing a rotary microtome, sections of uniform thickness were cut, forming ribbons that were then mounted on gelatin-coated slides. For each sample two slides sections were prepared for evaluation. The staining process involved deparaffinization using xylene, followed by hydration using descending alcohol grades and subsequent staining with hematoxylin and eosin. After dehydrating with ascending alcohol grades and clearing with xylene, the sections were cover-slipped with DPX (Distyrene Plasticizer Xylene).

To maintain objectivity, the slides underwent coding and blinding before histopathological assessment by a pathologist to reveal insights into tissue composition and characteristics.

## Results and discussion

### HPLC method development and stability of drugs

**Fig. 1:**
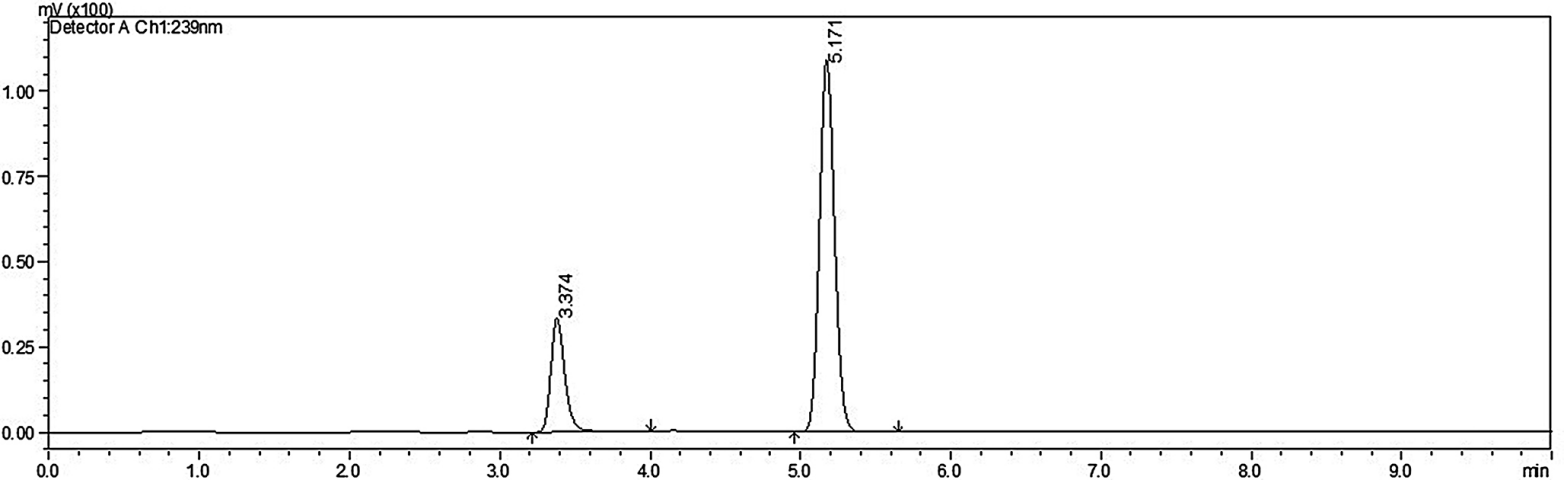
HPLC chromatogram for MOX (3.3 min) and DEX (5.1 min) at 239 nm wavelength, run in 50:50 acetonitrile: buffer

The drugs were analyzed using RP-HPLC. The retention time was found to be 3.3 min and 5.1 min for DEX and MOX respectively in the combination setup (Fig. 1). For the individual drugs, the retention time was 4.2 min for both (Supplementary Fig S2 and S3). The drug concentration in the dilutions prepared from the stock was quantified using the developed method. The standard graph plotted was later used to analyze the drug loading and in vitro release profile (Supplementary data Fig S4).

**Fig. 2:**
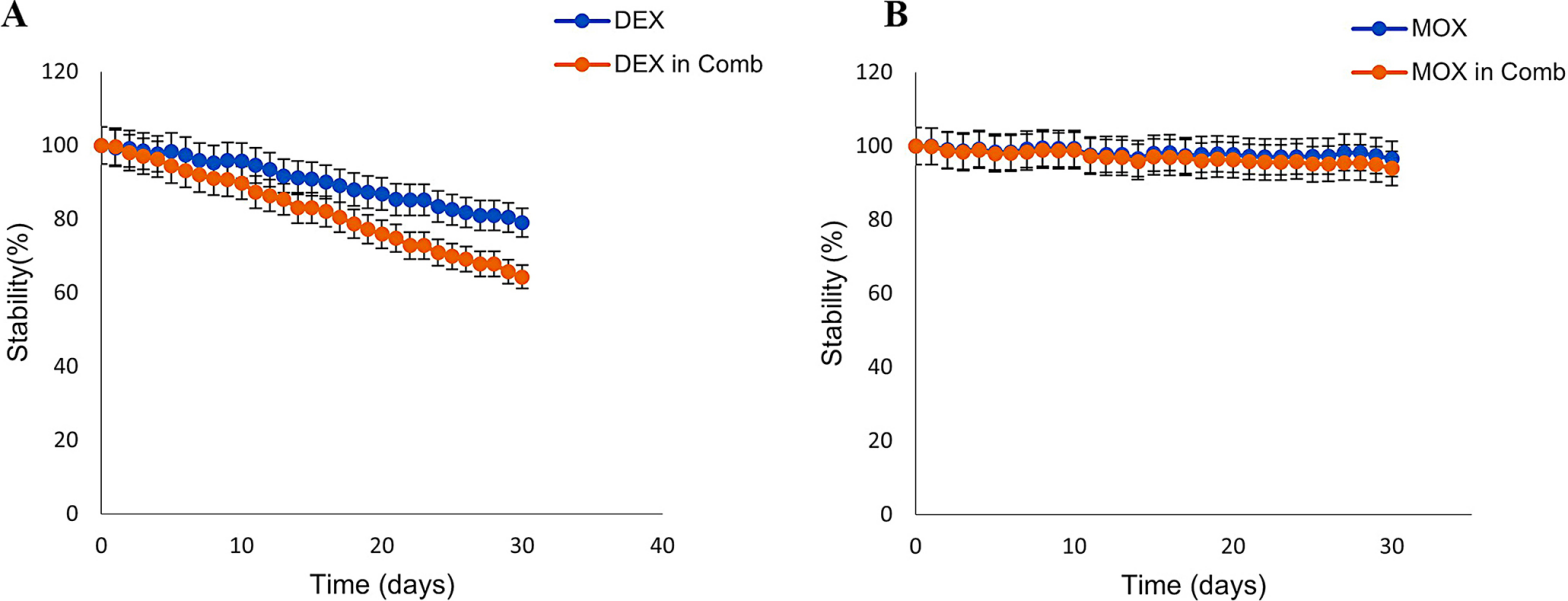
Stability profile of DEX (**A**) and MOX (**B**) individually and in combination in phosphate buffer of pH 7.4 kept at 37℃, analysed for a period of 30 days using RP-HPLC

During the stability analysis, it was noticed that MOX was relatively stable with less than 10% degradation observed in 30 days. Gradual degradation was observed in the DEX sample (Fig. 2A). While the degradation was comparatively more pronounced when DEX was placed in combination with MOX with 35% degraded by day 30 (Fig. 2B). This is probably due to interaction between the drugs in liquid phase. The PLGA matrix has a protective effect on the drugs from preventing degradation inside the implant. The degradation is initated only on exposure to the buffer [34]. This protective effect ensures the stability of the drugs within the implant as it remains inside the PLGA matrix. The aquoues humor turnover is 1-1.5% of the volume per minute, thus there is a complete turnover of of the aqueous humor for about every 100 min [35]. The release from the implant is in controlled and sustained manner and once it releases from the implant, it will be cleared in less than a day inside the eye. Additionally, we are also investigating potential drug-drug interactions. Though there is some interaction between the drugs but degradation of DEX in first 24 to 48 h of interaction with MOX is negligible. The stability studies were conducted for a period of 30 days was guided by the recommendations provided in the All India Ophthalmological Society (AIOS) guidelines, specifying application of antibiotics for 2 weeks and anti-inflammatory drugs for 4 to 6 weeks. Given that moxifloxacin release was completed around 22 days and considering the stability of the drugs within the implant, a 30-day stability study period was observed.

### Implant preparation and characterization

**Fig. 3:**
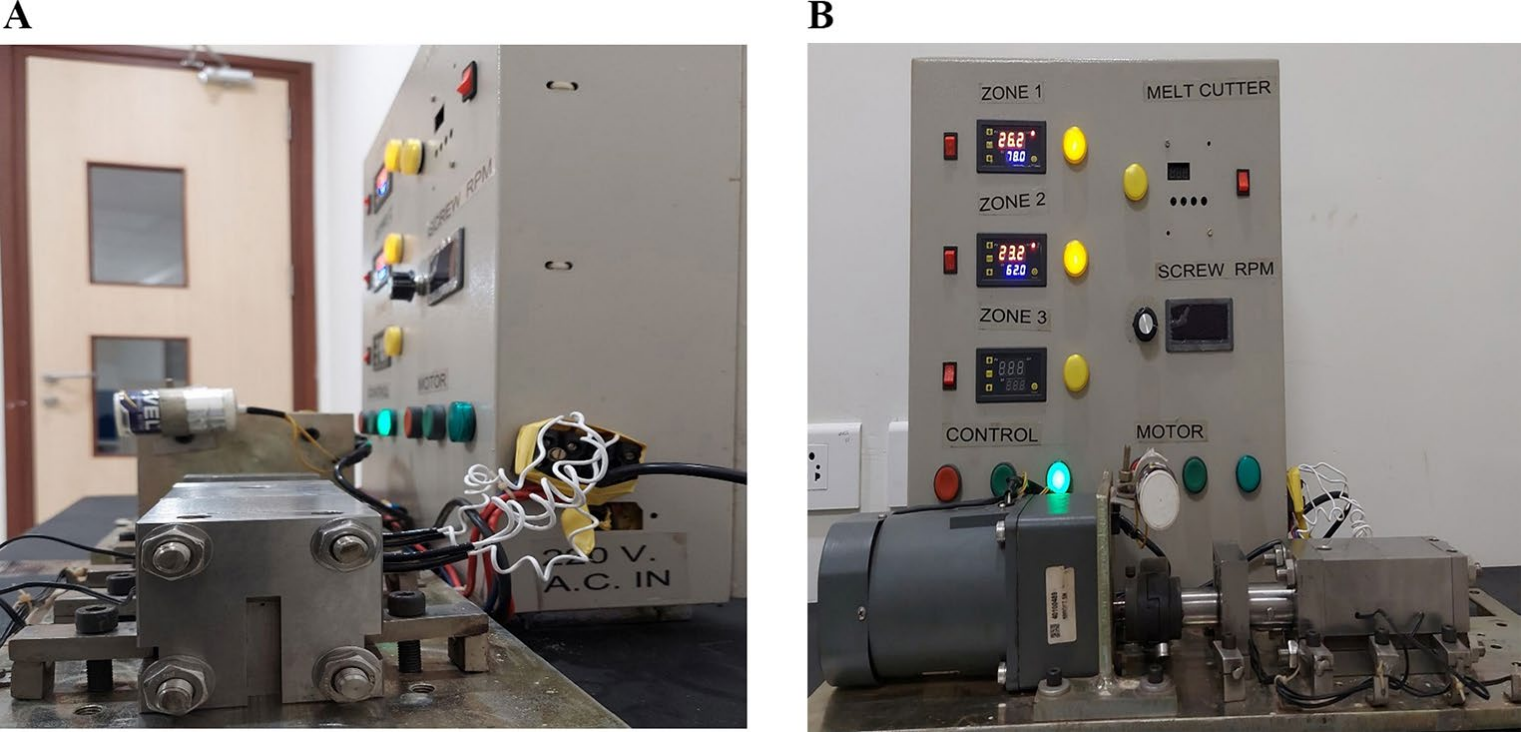
The custom made single screw hot melt extruder (**A**-side view), (**B**-front view)

The implants were prepared using a single screw hot melt extruder by setting the heating to a temperature above the glass transition temperature of PLGA (Fig. 3A&B) [36]. Hot-melt extrusion emerges as a viable approach in crafting biodegradable implants, proving its effectiveness through its continuous and industrial-friendly process. Its distinct advantage lies in eliminating the requirement for solvents or water, setting it apart from methods reliant on solvents [37]. One of the limitations faced while using the extruder was the inconstistency in the size of the filament obtained. In order to achieve consistent drug loading with minimal variation, we maintained a constant weight, enabling us to attain the desired level of drug loading (Table 2).

**Table 2:** Dimensions and weight of implant

Sl.No	Sample type	Diameter (mm)	Length (mm)	Weight (mg)
1	Combination	1.13	1.04	1.6
2	Combination	1.16	1.09	1.6
3	Combination	1.19	1.1	1.6
4	DEX	1.05	1.03	1.6
5	DEX	1.04	1.07	1.6
6	DEX	1.11	1.08	1.6
7	MOX	1.09	1.01	1.6
8	MOX	1	1.05	1.6
9	MOX	1.07	1	1.6
10	PLGA	1.14	1	1.6
11	PLGA	1.09	1.02	1.6
12	PLGA	1.10	1.01	1.6

**Fig. 4:**
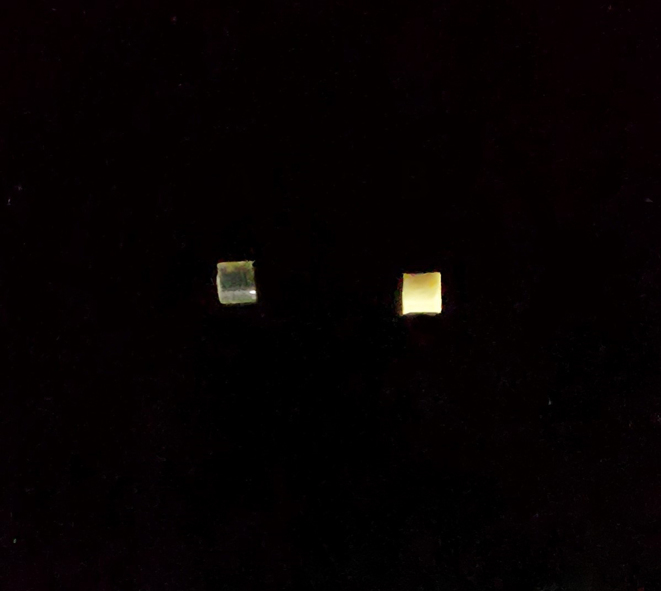
Implants- Plain PLGA(left) and DEX-MOX PLGA (right)

The filament was chopped to obtain implants weighing 1.6 mg each (Fig. 4). The combination implant had 270.3 ± 23 µg of DEX and 153.4 ± 12 µg of MOX per implant. DEX individual implant contained 268.8 ± 9 µg of the drug per implant and MOX individual implant contained 143.3 ± 2 µg of the drug per implant. The variation in drug loading among the three batches of prepared implant was found to be 3.1% for DEX and 8.4% for MOX.

The amount of drug to be used for the preparation of implant was calculated based on the total drug utilised during the post-operative care while using eye drops, drug loading in established ocular implants and works by Yan et al., and Chennamaneni et al. While calculating the amount of drug utilized with eye drops, one drop was considered as 50 µl, and the values of 1% and 5% of the total drug used were taken, as these are considered indicative of the bioavailability of the drug upon application of eye drops. Yan et al. [17] developed a hydrogel system incorporated with DEX, MOX and Genistein and Chennamaneni et al. [20]developed an implant by compression of DEX loaded PLGA microparticles. Both studies have highlighted the use of approximately 100–300 µg of DEX and 200 µg of MOX in developing drug delivery systems intended for insertion into the capsular bag to manage post-cataract surgery complications though in vivo studies were not conducted in the work by Yan et al. Despite the lower quantity of drugs loaded into the implant in our study compared to the quantities reported in previous research, our findings indicate a notable therapeutic effect. Notably, the amount of DEX integrated into PLGA implant Surodex is significantly less than what was utilized in our study, but it is essential to acknowledge that the drug release profile of Surodex lasts only for 7 days [38].

**Fig. 5:**
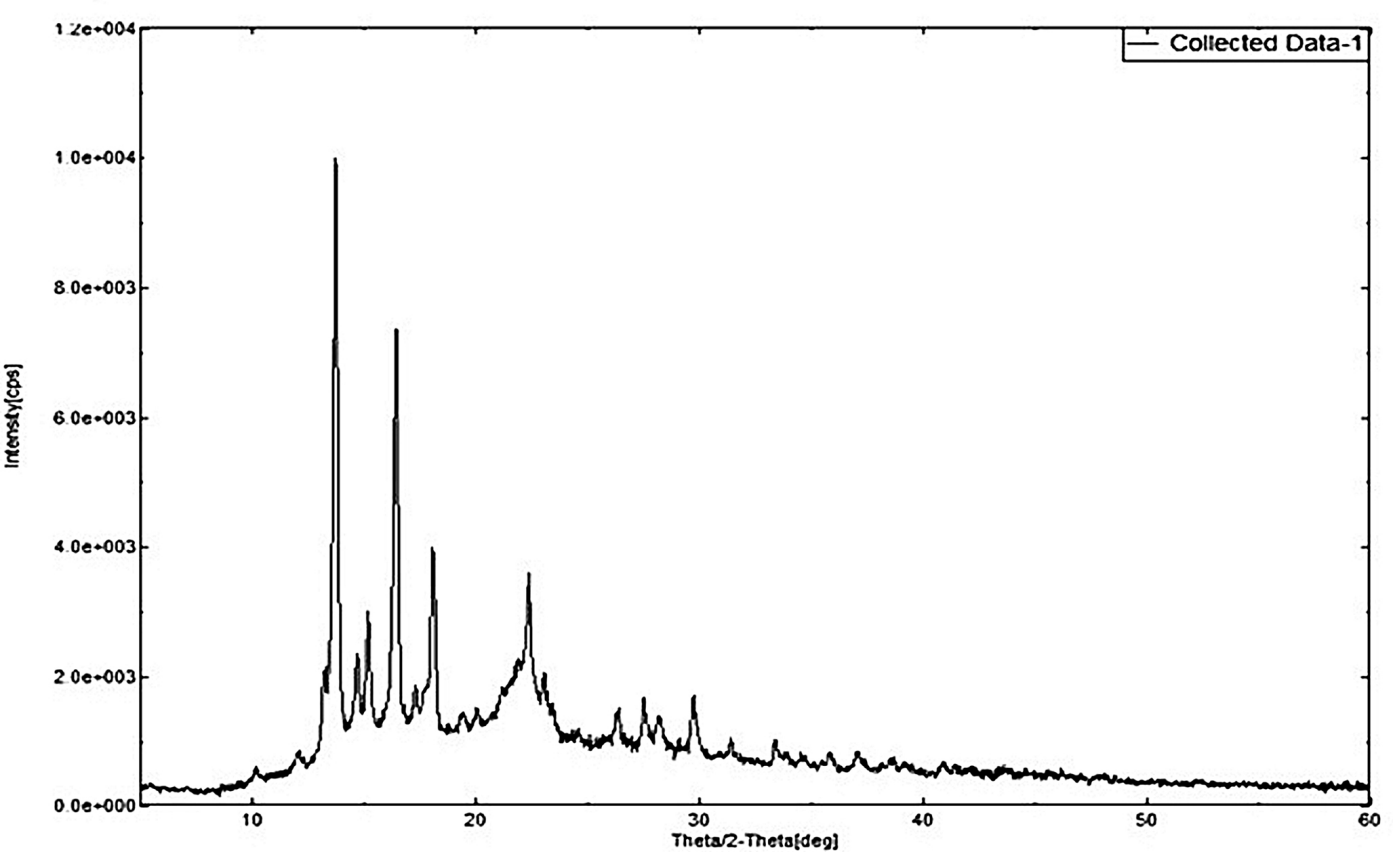
XRD of DEX showing sharp peaks indicating the crystalline nature of the drug

**Fig. 6:**
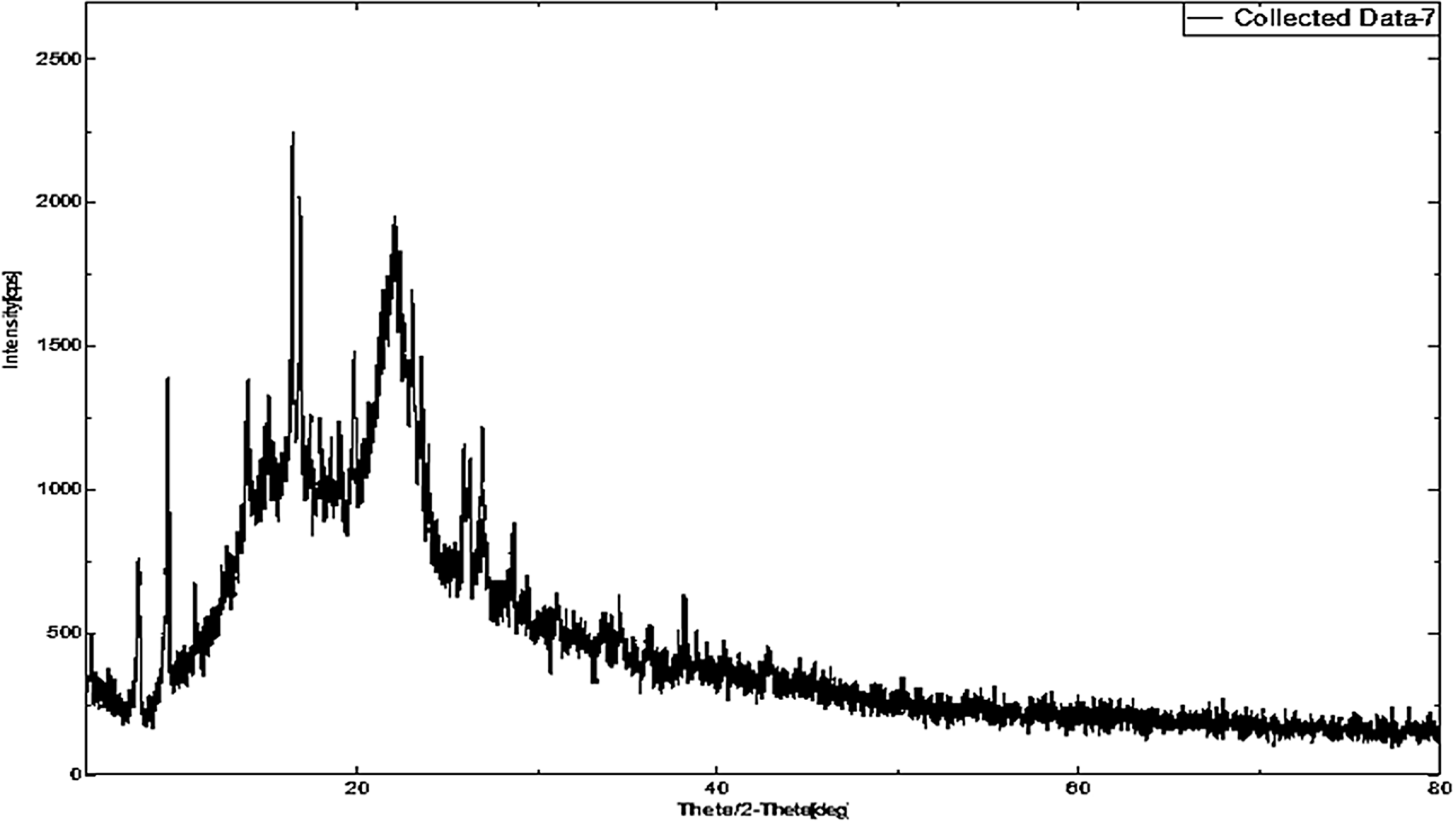
XRD of MOX showing sharp peaks indicating the crystalline nature of the drug

**Fig. 7:**
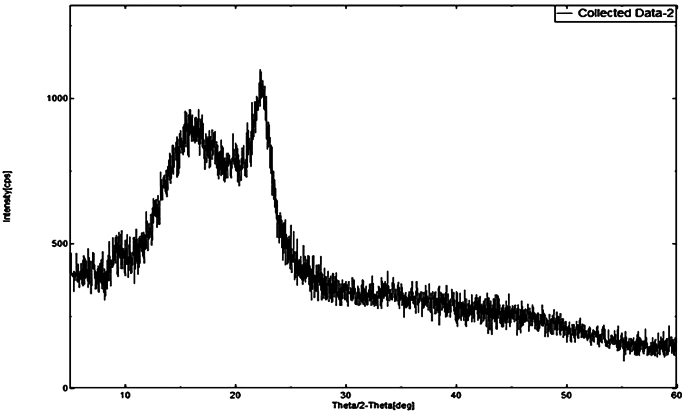
XRD of PLGA showing broad peaks indicating the amorphous nature of the polymer

**Fig. 8:**
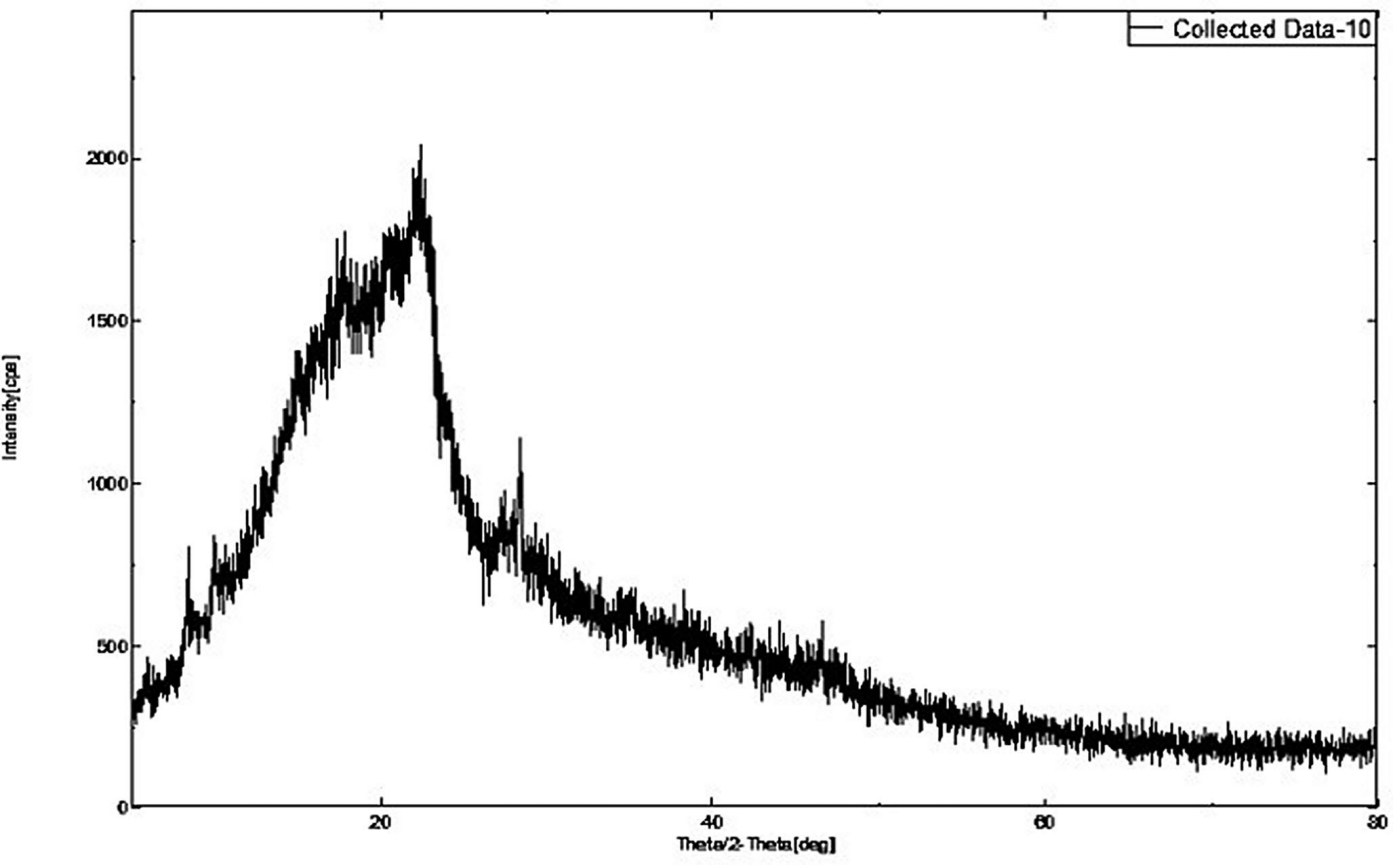
XRD of combination implant. The image shows broad peaks indicating amorphous nature, from which it can be inferred that the polymer masks the characteristic peaks of the drugs DEX and MOX and the drugs are uniformly distributed throughout the implant

XRD patterns were generated for DEX (Fig. 5), MOX (Fig. 6), PLGA (Fig. 7), and a combination of drug-loaded PLGA implants (Fig. 8) to understand the crystallinity of the developed product. The sharp peaks observed in the XRD patterns of DEX and MOX shows the crystalline nature of the two while the lack of the same in the XRD pattern of PLGA tells its amorphous nature. Characteristic broad peaks of amorphous nature were obtained on analysis of the drug loaded implant. This infers that the drugs are homogenously dispersed within the polymeric matrix.

**Fig. 9:**
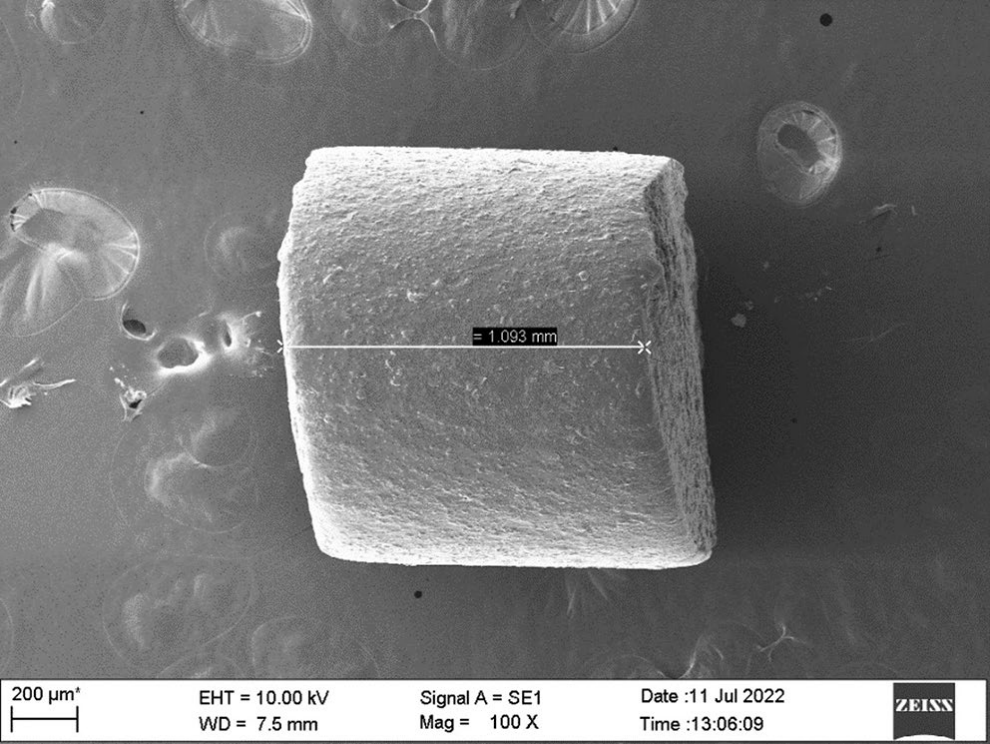
SEM image of the implant showing the implant length of 1.093 mm

**Fig. 10:**
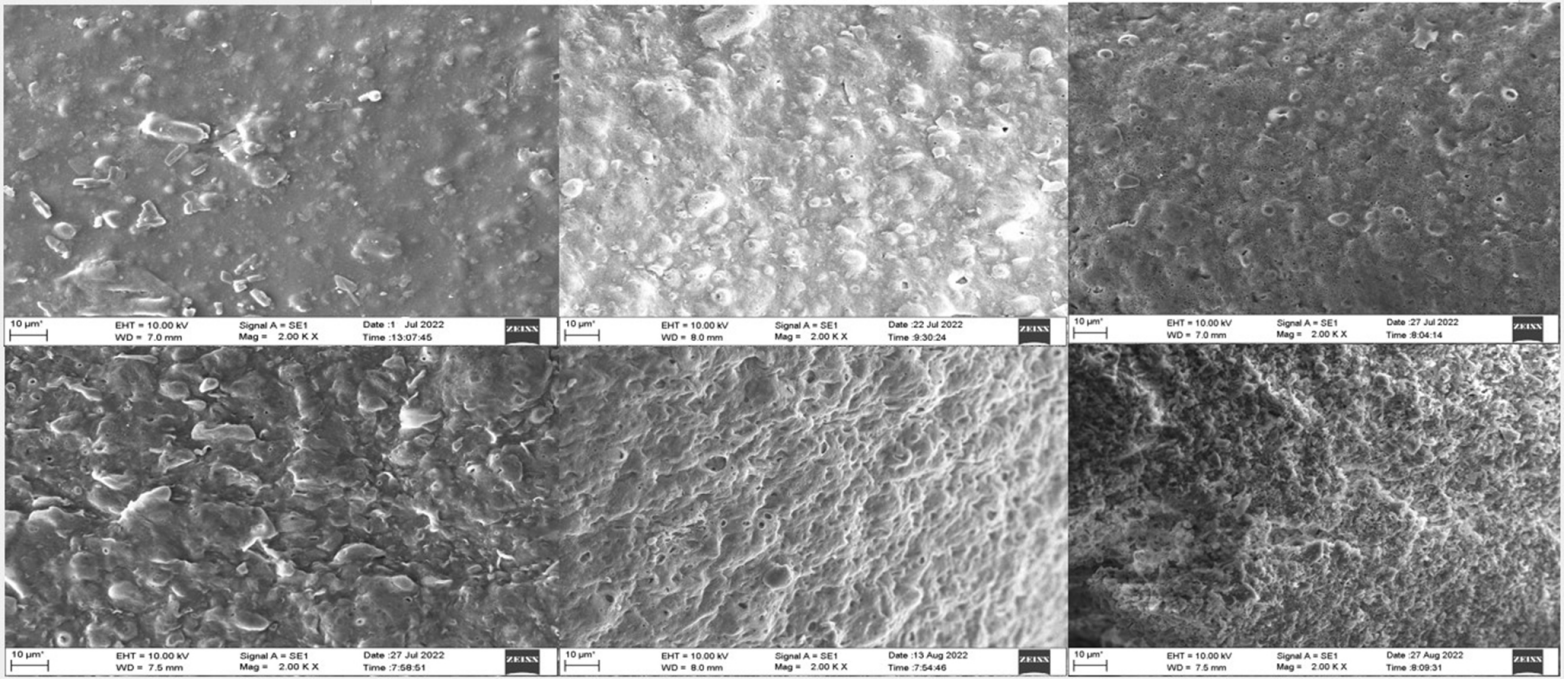
SEM image of the implant in phosphate buffer (pH 7.4) Row 1 left to right day − 0,7,21. Row 2 left to right- day28, 35,42. There is a gradual degradation as time passes when implant is placed in the buffer which can be observed in the increase in surface unevenness and porosity within the implant

Figure 9 shows the SEM image of the dual drug loaded implant. In Fig. 10 it can be observed that the surface turns more uneven over the course of the study. PLGA is a bulk eroding polymer [39], and the eroded areas seen on the surface show the degradation of the polymer. The primary mechanism behind drug release in PLGA polymer stems from a dual process involving the infiltration of water into its matrix and the gradual erosion of the bulk due to the hydrolysis of co-polymer chains [22]. On exposure to the aqueous medium, the water enters the system and initiates the degradation. The breakdown of PLGA into lactic and glycolic acid which resultes in the creation of an acidic environment, intensifying the autocatalyzing nature of the PLGA polymer [40]. When the drug is exposed to the penetrating water it either dissolves/diffuses out of the polymer matrix [41]. As the duration of exposure to the aqueous medium increases, the degradation is higher, as evidenced by the increased porosity observed on the implant.

### In vitro drug release

**Fig. 11:**
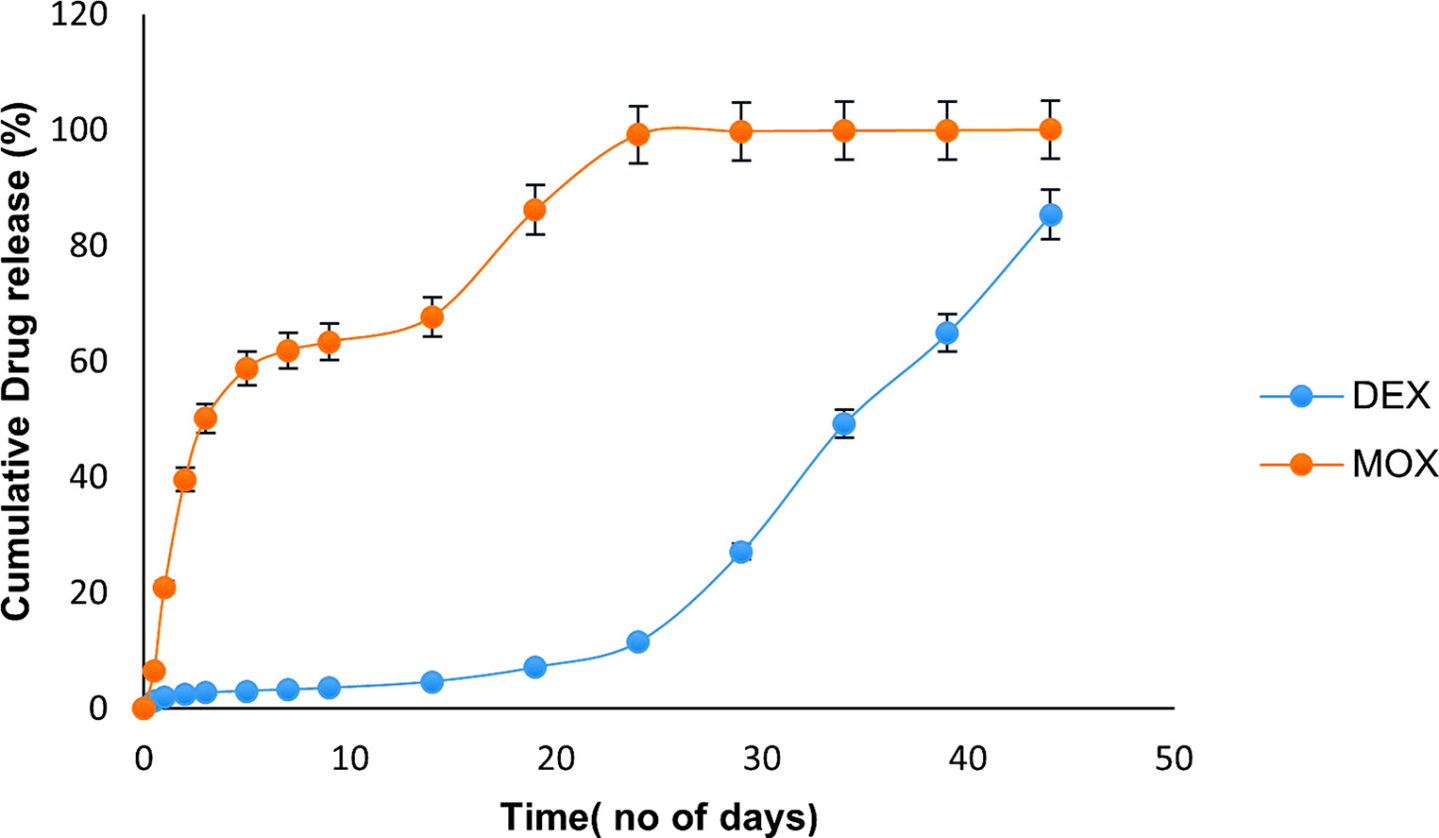
Release profile of DEX and MOX in combination implant when kept in phosphate buffer of pH 7.4 at 37℃. MOX releases at a faster pace and completes the release in about 20 days, while a lag is observed in the release of DEX in the first 15 days and the release extends to over a month

The in vitro release profile is as shown in Fig. 11. It was observed that MOX was released at a faster pace than DEX in the combination implant. There was a release of about 22% of MOX in 24 h while for DEX it was 2%. A similar trend was observed for the individual drug implants (Supplementary data Figs S5 and S6). There was not much variation in the release profile of the drugs from the implant when loaded individually and in combination. According to the AIOS guidelines, for the first fifteen days antibiotic should be administered with an anti-inflammatory drug, and next fifteen days only an anti-inflammatory drug should be administered through eye drops [6, 31]. We obtained a similar release trend of the drugs from the developed implant.

The PLGA polymer is used as the matrix. It is a biocompatible, biodegradable, and provides a method for controlled drug release for various time intervals [26]. Variations in the lactic acid: glycolic acid ratio, the molecular weight of the polymer, and the type of end group can help manipulate the drug release kinetics [20, 21]. DEX follows a triphasic release from PLGA implant which involves small burst release in the first 24 h which was about 2% which amounts to approximately 2 µg in this study, then a lag phase for about 15 days and then drug release increases from 3rd week onwards (Fig. 11). The initial burst release can be attributed to the loosely adhrerd DEX on the surface of the implant which is uncoated by the polymer matrix that can be easily released on exposure to water [42]. After the initial burst release in the first 24 h, the lag phase is noticed in the release of DEX which is approximately 12% of the total drug. This could be due to the hydrophobic nature of both the polymer and the drug which limits the exposure of drug to the buffer [25]. After the lag phase, a substantial increase in the release of DEX is noticed upto the completion of the drug release. This is because by this time there is sufficient degradation of the polymer and improved uptake of buffer into the matrix allowing the dissolution of the drug. Eye drops contains 1% DEX, each drop is considered 50 µl [43] and bioavailability of the drug on topical application is 1–5% [10]. In the first 24 h, the calculated amount of therapeutic drug (eye drop application 6 times a day) reaching the site of targeted tissue will be around 0.3–1.5 µg. In first 15 days, about 12% release of DEX occurced from the implant as observed in the in vitro studies. The drug quantities released from implant in this 15 days is within the cumulative amount of 1–5% bioavailability when eye drops are used for the same duration which is between 4.5 and 22.5 µg. From this we can conclude that there is no significant impact of the lag phase in the recovery of the rabbit. This triphasic release of DEX from PLGA implant has been reported for the intravitreal imlant Ozurdex [28, 42]. The difference in the release profile of DEX between the developed implant and Ozurdex can be attributed to its composition. The composition ratio of drug to polymer in Ozurdex consists of 60% DEX, 30% acid-terminated and 10% ester-terminated 50:50 PLGA (7-17KDa) [44]. However, in the fabricated implant, the ratio is approximately 17% DEX, 9% moxifloxacin (MOX) and 74% ester-terminated 50:50 PLGA (65-95KDa). The polymer drug ratio can also affects the release profile and the use of lower molecular weight and acid terminated PLGA increases the rate of degradation of the polymer [25]. Thereby, there is a shorter lag time and the release is completed faster in Ozurdex with the release being completed in about 30 days. The fabricated implant used in this study has higher molecular weight of PLGA and encapsulated much lesser amount of drug, leading to an increased lag time and longer release period. The release of DEX from the prepared implant is independent of the presence of MOX and a similar trend is observed in the release profile of the DEX only implant. The similar type of release was observed with thin film strip of PLGA encapsulated with DEX wound around the optic of IOL [19]. The faster release of MOX as noticed in the in vitro release profile may be because of the pronounced osmotic effect due to its hydrophilic nature [21].

### In vivo study

**Fig. 12:**
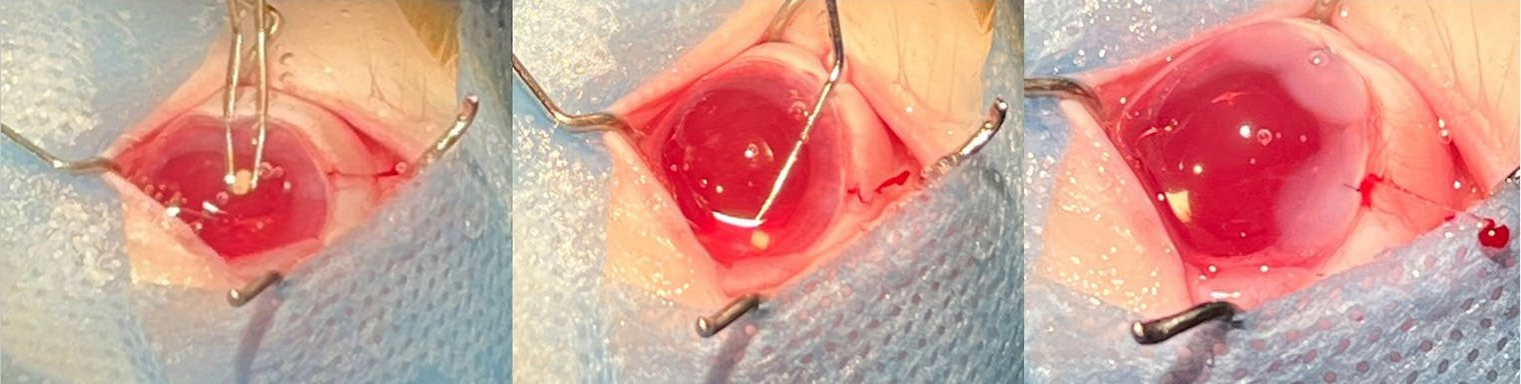
Implant insertion and positiong in the eye during surgery– left to right- Insertion, Position adjustment, Post adjustment in the capsular bag

**Fig. 13:**
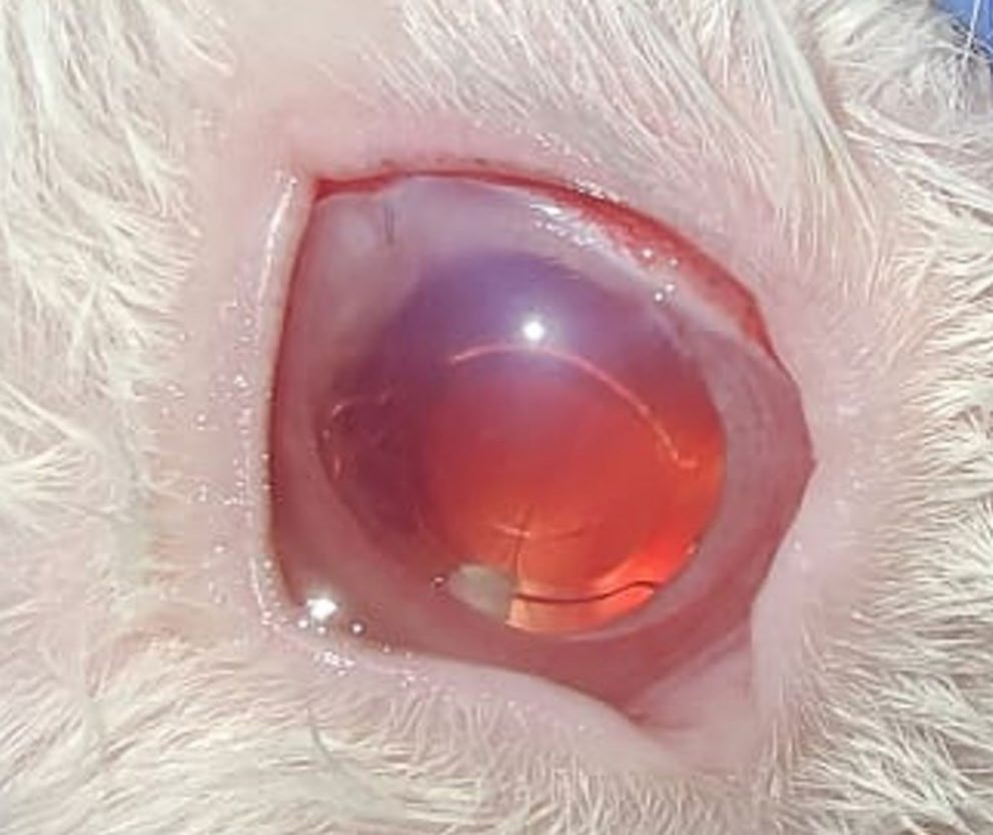
Implant in the capsular bag of the eye after surgery on day 1

**Fig. 14:**
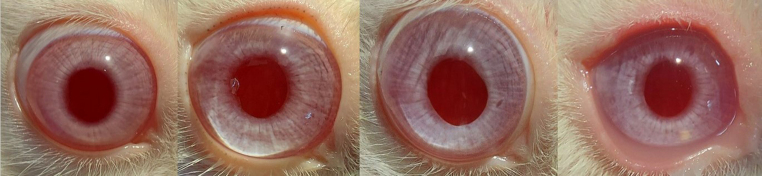
Operated eye at 6 weeks– from left to right- Normal control, Positive control, Sham control, Test. The implant can residue can be seen in the test sample behind the iris

Rabbits eyes were chosen as a model for this study condering the various anatomical similarities between human eye and rabbit eyes [45]. A total of 12 rabbit eyes underwent cataract surgery and out of this four of them received the dual drug loaded implant. The implant was inserted into the capsular bag after the placement of the IOL (Fig. 12). The slit-lamp observations revealed that there was no significant anterior chamber reaction in all groups for the majority of the study period. But we had observed a small anterior chamber reaction on day 1 after surgery which was there in almost all operated eyes but was resolved in the subsequent days. No displacement of the IOL was observed. It was difficult to spot the implant during the weekly observations, probably due to the reduction in size, degradation of polymer and increased transparency of the implant due to release of drugs. The implant was in the capsular bag inferiorly in most cases (Figs. 13 and 14). In one case in the test group, the implant had stuck to the center of the bag while in another case it had migrated to the anterior chamber. IOP measurement were taken every week post surgery (Table 3). An increased IOP was observed in the test group during the period of study which may be due to high DEX concentration in the eye as observed in the in vitro release profile. Ocular hypertension or elevation of IOP due to application of steroids have been reported to be dosage and time dependent [46, 47]. Concerning the elevated IOP, glaucoma due to the long-term use of steroids is a known complication [48]. This can be managed by making adjustments to the steroid load during implant preparation. Insertion of the implant in the capsular bag will allow the bidirectional flow of drugs, increase the drug availability to the local tissues and the sustained drug release will turn improve the clinical outcome [49]. As the implant is biodegradable, the insertion of the implant during surgery helps to combine surgery and treatment without the need for surgical removal of the implant once the drug reservoir is exhausted [50].

**Table 3:** IOP measurements in rabbit

Time	Intraocular Pressure (mm Hg)			
	Normal control	Positive control	Sham control	Test
Week1	25.5±3.5	27.5±5.5	28.5±4.5	31.5±3.5
Week2	22±4	26.5±7.5	25±2	38±2
Week3	30	31.5±1.5	26±1	43.5±0.5
Week 4	23.5±6.5	31±1	28.5±1.5	33±3

### Histopathology

**Fig. 15:**
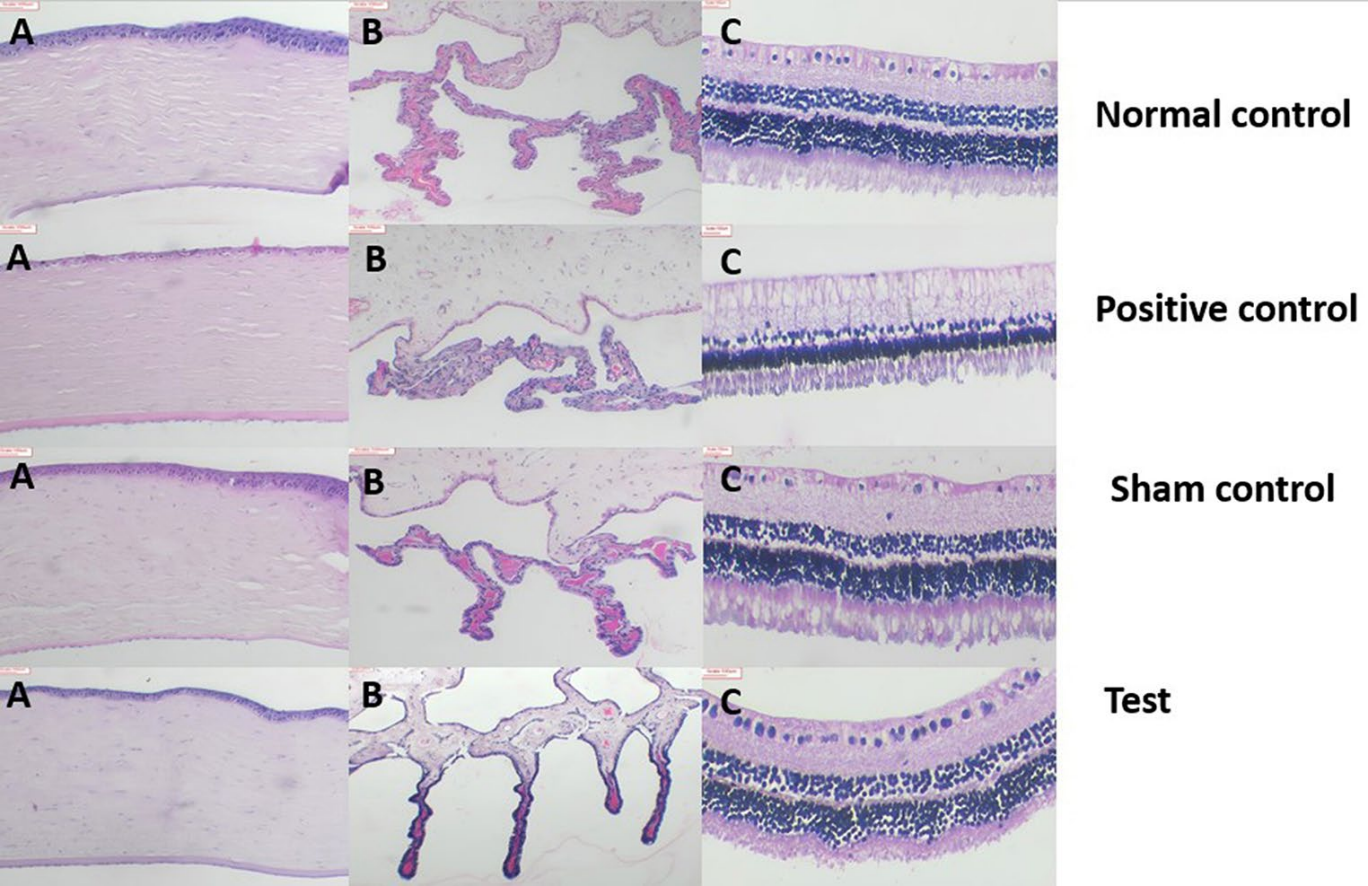
Histology of ocular tissues of right eye at 6 weeks (**A**) Cornea, (**B**) Iris and ciliary body, (**C**) Retina. No significant pathology was observed between the various study groups (Scale bar − 100 μm)

After the observation period, the eyes of the rabbits from all groups were enucleated and taken for histopathology studies. On gross examination of the operated eye under the microscope, it was observed that the cornea was clear, the iris was normal, capsular bag was intact and IOL was in the capsular bag. The histopathology results are based on the report given by the pathologist. There was no gross histopathological variation observed in the samples of unoperated eyes. No significant pathology was observed in samples of the normal control group. Focal edema and a few chronic inflammatory cells were observed in the cornea of the samples from the sham control group. Edema and mild chronic inflammation of the cornea were observed in the samples of the positive control group. In the test group, the cornea was normal. Edema and mild chronic inflammation in the iris and the ciliary body was observed. One sample of the test group showed congested blood vessels in the choroid. The retina appeared normal with no significant pathology. Any other differences seen in the images are artifactual, and occurred during the preparation process of the specimens. The results revealed the biocompatibility of the implant at the site of insertion (Fig. 15). Images of the cornea, ciliary body and retina of all the samples are given in the supplementary data (Supplimentary data FigS7 a, b and c).

## Conclusions and future work

The delivery of drugs via eye drops encounters challenges because of numerous ocular barriers. This study focuses on the creation of a dual drug-loaded biodegradable implant to manage post-cataract surgery inflammation and infection. We have demonstrated that the developed implant, when positioned in the capsular bag, effectively addresses the limitations of topical drug administration. The drugs DEX and MOX were concurrently released from the implant for a period extending beyond 30 days, achieving therapeutic efficacy. The release pattern of drugs from the implant follows a similar trend of the prescribed dosage regimen. During the initial week, there is a delay in DEX release, and a marginal increase in IOP was observed in the test groups. Hence, future work will involve optimizing the drug-loading and the release profile of the implant by exploring different polymer variations. Additionally, efforts will be directed towards ensuring the consistency in the size of filament to meet the requirements of bulk production. We strive to work towards translating this preliminary preclinical data to the next level of creating a Pharmaceutical product.

## Electronic supplementary material

Below is the link to the electronic supplementary material.

Supplementary Material 1

## Data Availability

The data generated and analysed during the course of the study are available from the corresponding author on request.
