# Supplementary material for: Development of a biodegradable polymer-based implant to release dual drugs for post-operative management of cataract surgery
Source: Drug Deliv Transl Res. 2024 May 2;15(2):508–22. doi: 10.1007/s13346-024-01604-y (PMC11683021; doi:10.1007/s13346-024-01604-y)
Supplement: Supplementary file 1 — Supplementary Material 1 [file 13346_2024_1604_MOESM1_ESM.docx]

**DEVELOPMENT OF A BIODEGRADABLE POLYMER-BASED IMPLANT TO RELEASE DUAL DRUGS FOR POST-OPERATIVE MANAGEMENT OF CATARACT SURGERY**

Nayana E Subhash^1^, Soumya Nair^2^, Srilatha Parampalli Srinivas^3^, Nagarajan Theruveethi^4^, Sulatha V Bhandary*^2^, BharathRaja Guru*^1^


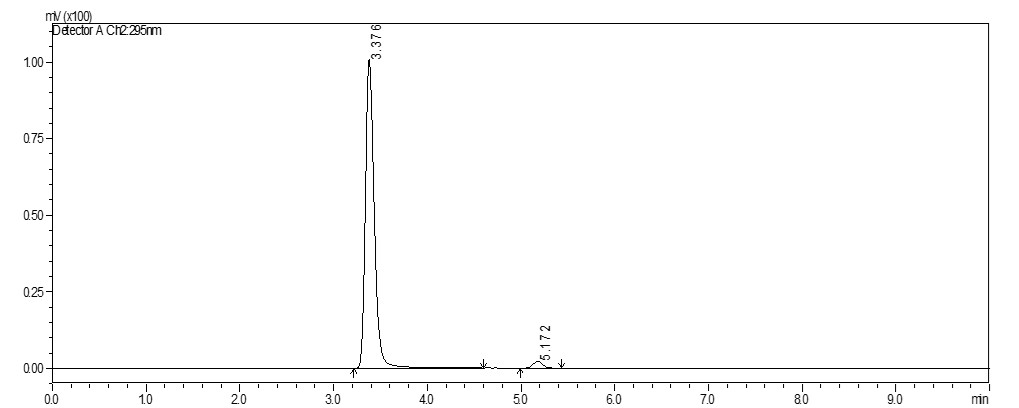


**Fig S1:** HPLC chromatogram for MOX (3.3min) at 295nm wavelength, run in 50:50 acetonitrile: buffer


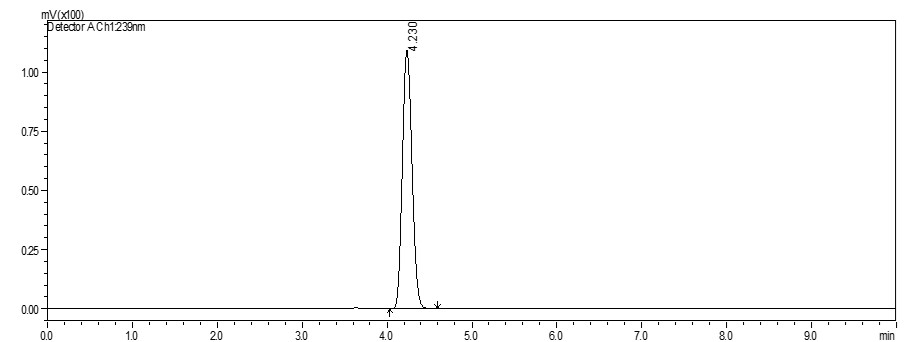


**Fig S2:** HPLC chromatogram for DEX (4.2min) at 239nm wavelength, run in 60:40 acetonitrile: water


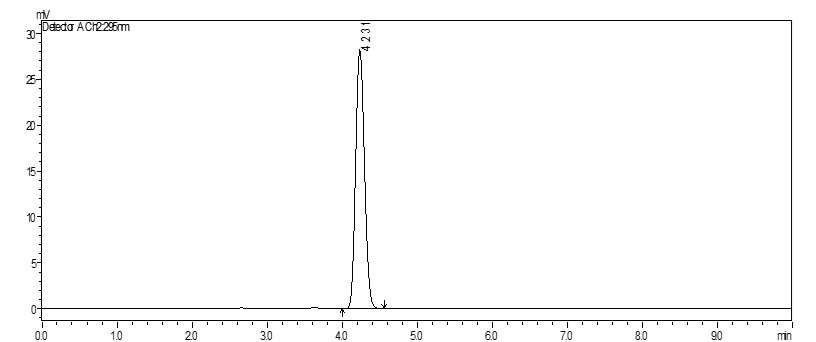


**Fig S3:** HPLC chromatogram for MOX (4.2min) at 295nm wavelength, run in 35:65 acetonitrile: buffer


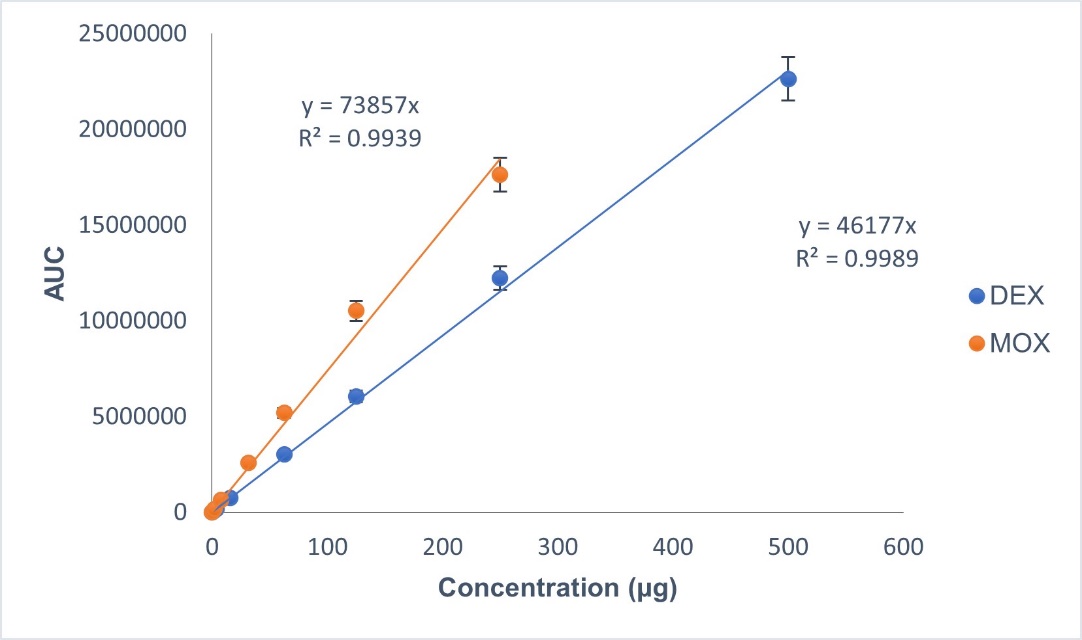


**Fig S4:** Standard graph for simultaneous estimation of DEX and MOX


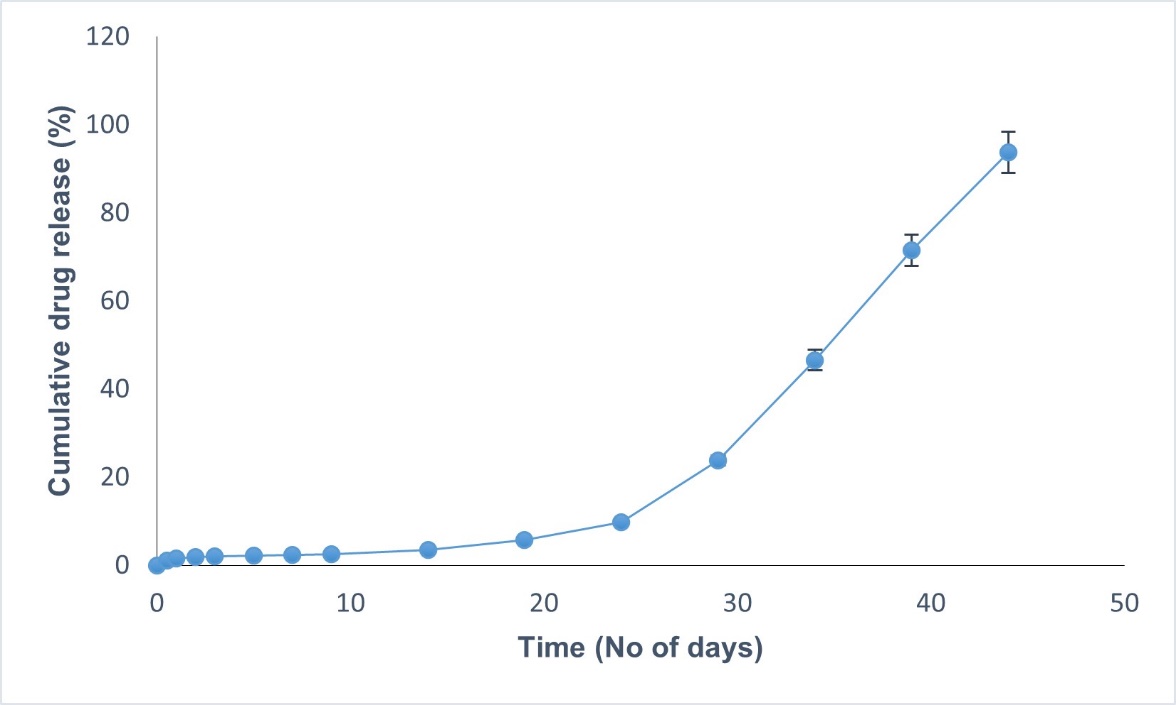


**Fig S5:** Release profile of dexamethasone in DEX only implant


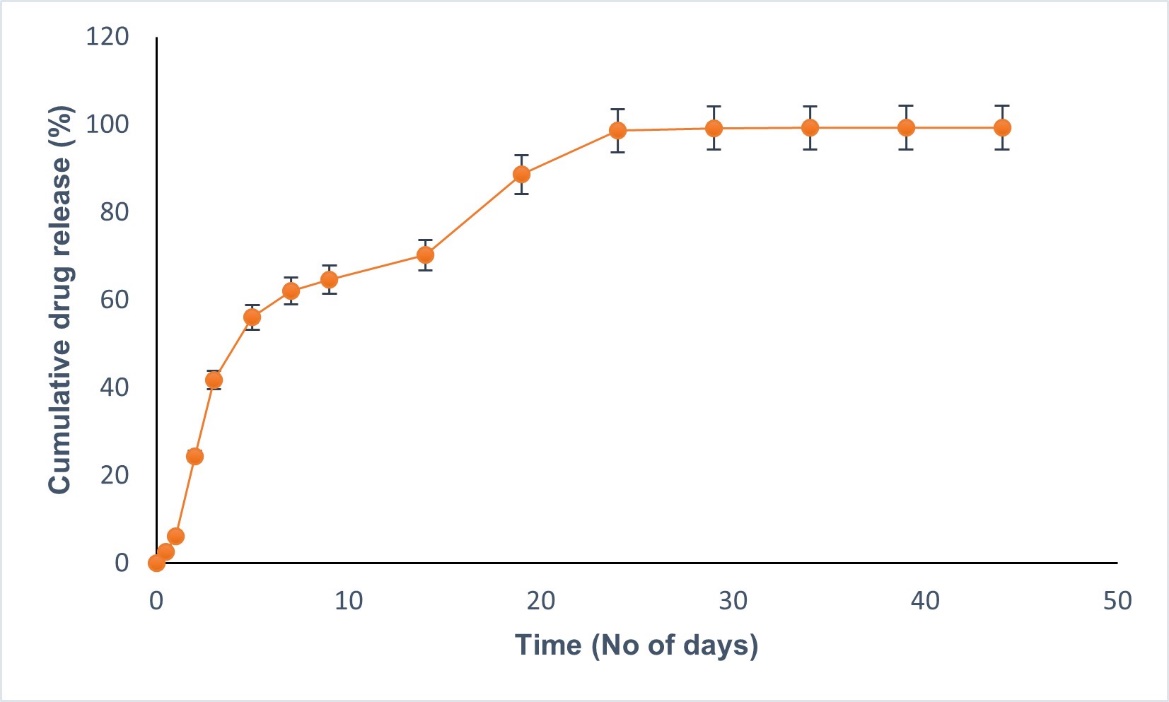


**Fig S6:** Release profile of moxifloxacin in MOX only implant


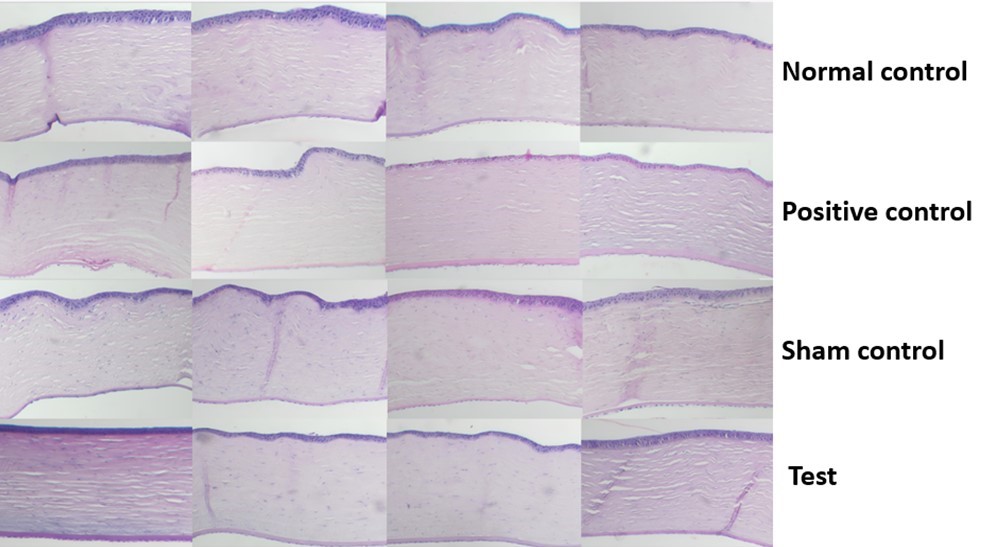


**Fig S7a:** Histology of cornea at 6 weeks (n=4)


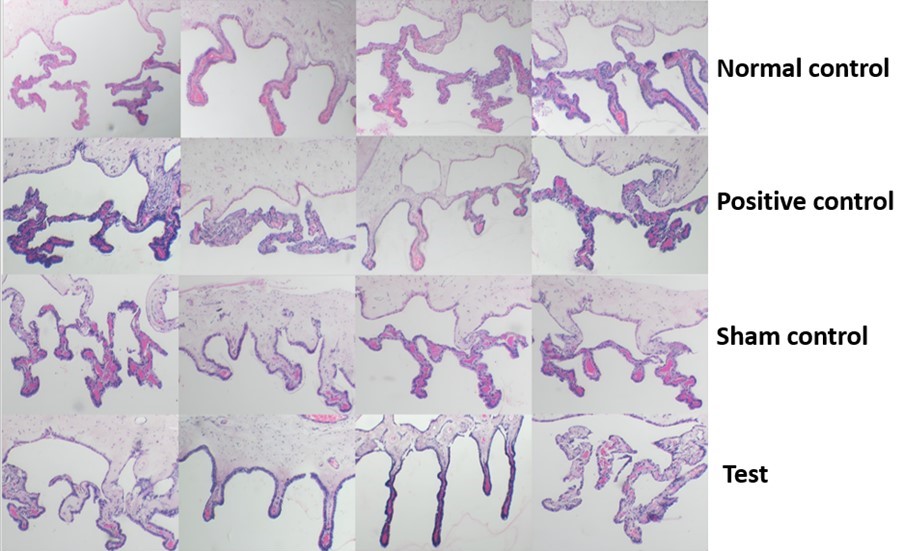


**Fig S7b:** Histology of ciliary body at 6 weeks (n=4)


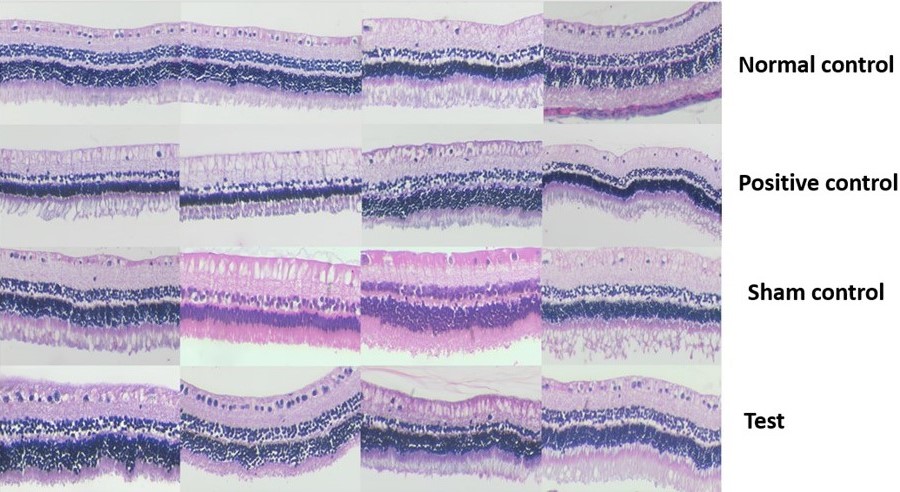


**Fig S7c:** Histology of retina at 6 weeks (n=4)
